# A Rare Case of Dysphagia with Palatal Tremor

**DOI:** 10.1007/s00455-020-10185-0

**Published:** 2020-09-03

**Authors:** Tao Xiang, Feng Wang, Yingyue Yang, Juan Li

**Affiliations:** 1 Department of Rehabilitation Medicine, Chengdu Second People’s Hospital, No. 10 Qingyunnan Street, Jinjiang District, Chengdu, 610017 Sichuan China; 2 Department of Neurology, Chengdu Second People’s Hospital, Chengdu, 610017 Sichuan China

## Case Presentation

A 77-year-old man with a known history of long-standing hypertension experienced a left cerebellar hemorrhage 9 months ago, then a right cerebellar hemorrhage 3 months prior to his presentation. The patient had noticeable dysphagia that required placing a nasogastric tube for nasal feeding. The standardized swallowing assessment revealed that he had an impairment in lip closure, head, and trunk control, pharyngeal reflex, as well as independent coughing. To observe the pathophysiological changes of the pharynx and larynx, a fiberoptic laryngoscope was inserted prior to the video fluoroscopic swallowing study (VFSS), which showed that there was no pathophysiological change. VFSS detected the rhythmic tremor of the soft palate and epiglottis, with residues displayed in the vallecula and pyriform sinuses.

**Fig. 1:**
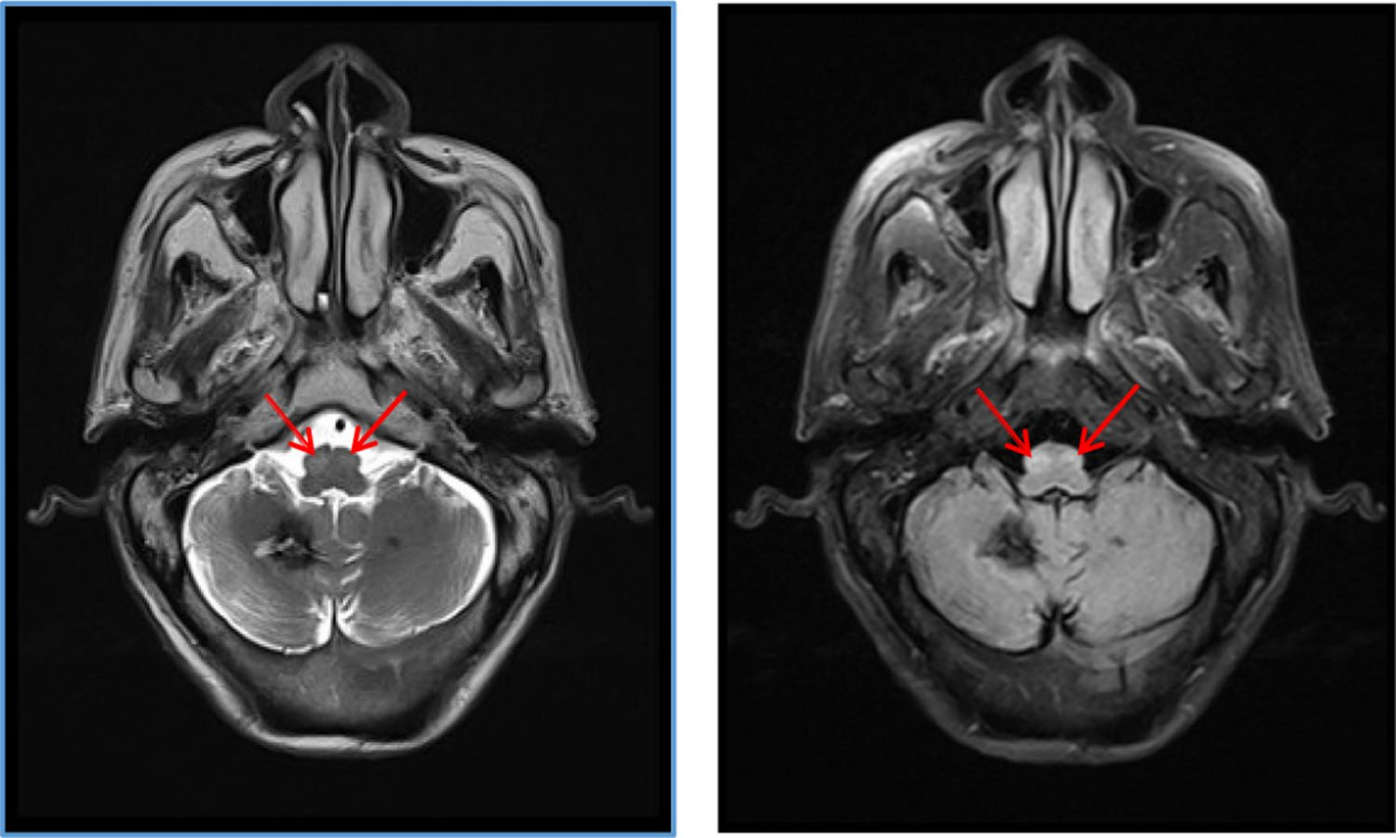
Axial T2 and FLAIR images: long T2 signal shadows were observed in the right cerebellum, and low-density circular shadows were observed around the lesion, with patchy long T2 signal changes in the left cerebellum. The area of the bilateral olivary nucleus was enlarged, with a longer T2-weighted signal change, and the T2 FLAIR image showed an increased signal change, the right ION was obviously larger than the left

Magnetic resonance imaging (MRI) depicted bilateral long T2 signal shadows in the cerebellum, enlargement of the bilateral olivary nucleus, with a longer T2-weighted signal change. The T2 FLAIR image demonstrated an increased signal change, with the right inferior olivary nucleus (ION) obviously larger than the left. (Fig. 1) (9 months after the left cerebellar hemorrhage, 3 months after the right cerebellar hemorrhage).

## Diagnosis

The patient presented with a palatal tremor (PT) after two episodes of cerebellar hemorrhage. MRI indicated bilateral hypertrophic degeneration of the olivary nucleus, especially on the left side. These findings taken together confirmed a diagnosis of hypertrophic olivary degeneration (HOD).

HOD is a rare neurological condition characterized by trans-synaptic degeneration that occurs secondary to focal lesions disrupting the normal function of the afferent fibers to the ION as part of the dentate–rubro–olivary pathways (Guillain–Mollaret triangle, GMT) [1]. Possible etiologies for this condition include infarction, toxicosis, trauma, surgery, tumors, vascular malformations or hemorrhage. In this case, the patient developed HOD secondary to cerebellar hemorrhage [2].

## Discussion

**Fig. 2:**
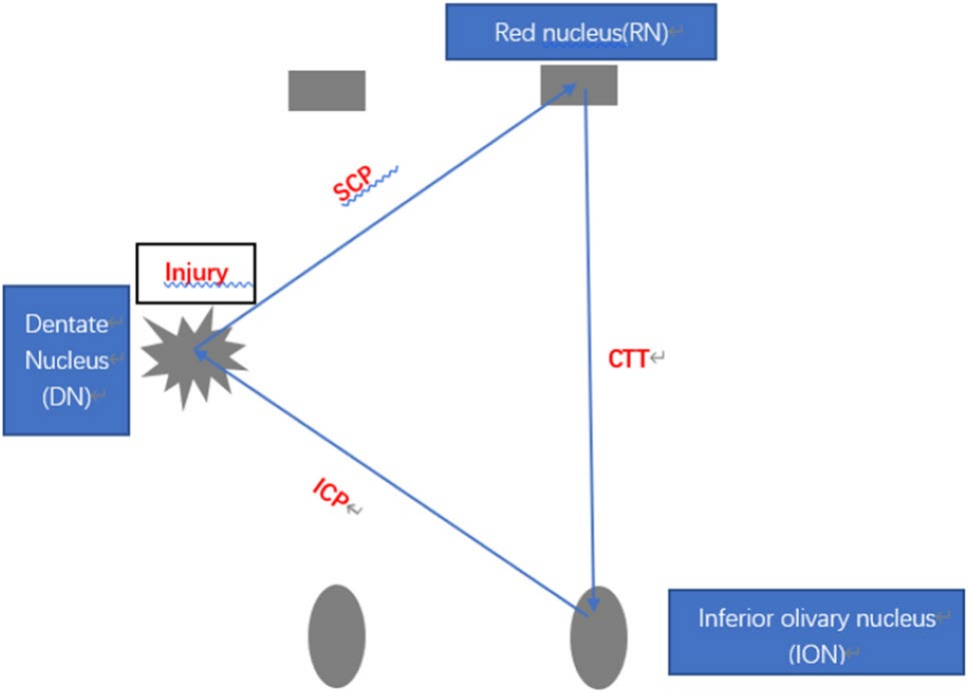
Guillain–Mollaret triangle

GMT is composed of the contralateral dentate nucleus (DN), the ipsilateral red nucleus (RN) and the ipsilateral ION. DN, RN, and ION form an afferent and efferent loop pathway. The fibers from the DN are crossed by the superior cerebellar foot and reach the contralateral midbrain red nucleus. The red nucleus sends fibers descending through the central tegmental bundle to the ipsilateral ION for relay. The ION is then emitted from fibers passing through the cerebellar inferior foot (ICP) to the contralateral cerebellar cortex and projected onto the DN, constituting the complete GMT [2] (Fig. 2).

When the primary lesion is located within the cerebellum, contralateral ION degeneration occurs, which is due to the decussation of the dentate-rubral fibers. A unilateral lesion in a cerebellar hemisphere usually causes contralateral HOD, and only a rare number of cases of bilateral HOD have been described with lesions affecting a single cerebellar hemisphere [2].

HOD is characterized by enlargement and increased T2 hyperintense signal of the ION [3]. The classic clinical presentation associated with HOD is a PT [4] which was observed in this patient. Dentatorubral tremors and ocular myoclonus are other associated movement disorders [4], but were not observed in this case. This muscle tremor may affect the patient's peristalsis, which is manifested as an incomplete epiglottic closure during swallowing, food residues in the vallecula and pyriform sinuses, leading to penetration-aspiration, resulting in dysphagia. The exact pathogenesis of the tremor is not clear, but the currently accepted hypothesis involves the interruption of GABAergic descending inhibition of the dentate–olivary tract during de-afferentiation of the triangle [5].

We treated the patient with electric stimulation, vocal cord movement and airway protection training for dysphagia, but the results were not satisfactory. Previous literature reports indicated that the medical treatment of PT is unsatisfactory and is often ineffective. some patients may respond to medications such as valproic acid, carbamazepine, clonazepam, tryptophan, trihexyphenidyl, or levetiracetam [6]. This patient was treated sequentially with propranolol, clonazepam, and levetiracetam, but his symptoms persisted.

## Conclusion

In this study, we report an unusual case of dysphagia following two episodes of cerebellar hemorrhage. Despite the fact that dysphagia after stroke is fairly common, the different etiologies and pathogenesis related to this condition should be identified, especially the rare causes of dysphagia. The patient is not responding well to treatment; thus, we need to follow up him for a long period of time to observe the changes in his condition.
